# Microelements as risk factors for cancer of the lung and larynx

**DOI:** 10.1186/1897-4287-10-S4-A2

**Published:** 2012-12-10

**Authors:** Katrzyna Jaworska-Bieniek, Satish Gupta, Katarzyna Durda, Magdalena Muszynska, Grzegorz Sukiennicki, Elżbieta Jaworowska, Tomasz Grodzki, Mieczysław Sulikowski, Piotr Woloszczyk, Janusz Wójcik, Jakub Lubiński, Cezary Cybulski, Tadeusz Dębniak, Marcin Lener, Steven A Narod, Ping Sun, Jan Lubiński, Anna Jakubowska

**Affiliations:** 1 International Hereditary Cancer Centre, Department of Genetics and Pathology, Pomeranian Medical University, Szczecin, Poland; 2 Postgraduate School of Molecular Medicine, Warsaw Medical University, Warsaw, Poland; 3 Department of Otolaryngology and Laryngological Oncology, Pomeranian Medical University, Szczecin, Poland; 4 Department of General Thoracic Surgery, Pomeranian Medical University, Szczecin, Poland; 5 Maxillofacial Surgery, Pomeranian Medical University, Szczecin, Poland; 6 Department of Toxicology and Molecular Pathobiochemistry, Pomeranian Medical University, Szczecin, Poland; 7 Women’s College Research Institute, Toronto, ON, Canada

## 

Selenium deficiency has been suggested by several studies to be associated with cancer risk. We conducted a case-control study in Szczecin, a region of northwestern Poland, on 86 cases of lung cancer, 87 cases of laryngeal cancer and an equal number of healthy controls. We studied the serum level of selenium and genotypes for four variants in four selenoprotein genes (*GPX1*, *GPX4*, *TXNRD2* and *SEP15*) and the odds of being diagnosed with lung or laryngeal cancer.

Among lung cancer cases, the mean selenium level was 63.2 µg/l, compared to a mean level of 74.7 µg/l for their matched controls (p < 0.0001). Among laryngeal cancer cases, the mean selenium level was 64.8 µg/l, compared to a mean level of 76.3 µg/l for their matched controls (p < 0.0001). Compared to a serum selenium value in the lowest of four categories (≤ 60 µg/l) a selenium level in the highest category (> 80 µg/l) was associated with an odds ratio of 0.10 (95% CI 0.03 to 0.34; p = 0.0002) for lung cancer and 0.24 (95% CI 0.10 to 0.59; p = 0.002) for laryngeal cancer. In four selenoproteins studied here we found a modest associations of genetic variants in *GPX1* and *GPX4* with lung and *TXNRD2* with laryngeal cancer risk.

We analyzed iron (Fe) level in serum of 77 lung cancer patients and 77 matched controls. We did not find difference in mean Fe level between cases and controls (1053.05 µg/l and 1059.39 µg/l). However, we found that Fe level in the lowest and highest quartiles was associated with a significant lung risk enhancement when compared to a serum Fe level in the middle quartiles (OR 0.3, p 0.02). We also observed that the relationship between the level of Fe and Se could be an important factor for lung cancer risk.

## Conclusion

1. Se is the strong marker of high risk of lung and laryngeal cancers and can be potentially used for detection of early cancers. Based on data showing higher Se supplementation as to be associated with lower cancer mortality (OR.55) Se supplementation should be considered to all patients with already diagnosed cancer.

2. Very low and very high Fe level seems to be associated with increased lung cancer risk

3. Most probably, for exact cancer risk assessment it will be necessary to analyze relationships between microelements, ie. Fe/Se ratio.

